# Development and external validation of a machine learning model based on preoperative nutritional status for predicting acute kidney injury after coronary artery bypass grafting

**DOI:** 10.3389/fnut.2026.1750814

**Published:** 2026-03-13

**Authors:** Zhaodi Wang, Jinghao Song, Yang Gao, Jiankang Zheng, Yuxia Qi, Jie Li

**Affiliations:** 1 Department of Internal Medicine, Qingdao Public Health Clinical Center, Qingdao, Shandong, China; 2 Department of Cardiovascular Surgery, The First Affiliated Hospital of Shandong First Medical University & Shandong Provincial Qianfoshan Hospital, Jinan, Shandong, China; 3 Department of Cardiology, Affiliated Hospital of North Sichuan Medical College, Nanchong, Sichuan, China; 4 Department of Anesthesiology, Affiliated Hospital of Jining Medical University, Jining, Shandong, China; 5 Department of Anesthesiology, The First Affiliated Hospital of Shandong First Medical University & Shandong Provincial Qianfoshan Hospital, Jinan, Shandong, China; 6 Center of Health Management, Qilu Hospital of Shandong University, Jinan, Shandong, China

**Keywords:** acute kidney injury, coronary artery bypass grafting, nutritional status, machine learning, risk prediction model

## Abstract

**Background:**

Acute kidney injury (AKI) is a common complication after coronary artery bypass grafting (CABG). Preoperative nutritional status may influence AKI risk, but its predictive value remains unclear.

**Methods:**

We retrospectively analyzed 811 CABG patients from two centers. Nutritional status was assessed using the Controlling Nutritional Status (CONUT) score, Prognostic Nutritional Index (PNI), and Geriatric Nutritional Risk Index (GNRI). Logistic regression and restricted cubic splines evaluated associations with AKI. The most predictive index, combined with key clinical variables selected via LASSO and Boruta, was used to build six machine-learning models. Model interpretability was assessed using SHAP, and a web-based calculator was deployed.

**Results:**

All three indices were independently associated with AKI, with PNI performing best (AUC = 0.617). The GBM model showed highest predictive performance with AUCs of 1.000, 0.978, and 0.905 in training, internal, and external validation sets, respectively. SHAP identified PNI, LVEF, and CPB as top contributors.

**Conclusion:**

Preoperative nutritional status, particularly PNI, is an independent predictor of AKI. An interpretable GBM model incorporating nutritional and clinical variables enables accurate individualized risk assessment.

## 1 Introduction

Coronary artery bypass grafting (CABG) remains a cornerstone surgical intervention for the management of severe coronary artery disease (1, 2). Despite advancements in surgical techniques and perioperative care, postoperative acute kidney injury (AKI) continues to be a frequent and serious complication, affecting approximately 19–28% of patients (3–5). The occurrence of AKI following CABG is associated with prolonged hospitalization, increased healthcare costs, elevated mortality rates, and a heightened risk of progression to end-stage renal disease (ESRD) (6–8). Consequently, early identification of patients at risk for AKI is critical to improving postoperative outcomes and optimizing perioperative management strategies.

Malnutrition, characterized by a reduction in body fat and/or muscle mass due to insufficient nutrient intake or impaired nutrient utilization (9), has recently garnered increasing attention as a potential modifiable risk factor for AKI. Previous studies have demonstrated that malnutrition exacerbates the risk of AKI in hospitalized patients, as well as in those suffering from sepsis, malignancies, or acute coronary syndromes (10–13). Moreover, emerging evidence suggests that malnutrition and frailty are independent predictors of AKI in patients undergoing CABG (14, 15). However, traditional nutritional markers such as serum albumin levels and body mass index (BMI) have inherent limitations in stability and comprehensiveness, which constrain their utility in accurately stratifying nutritional risk (16). To address these limitations, several objective and standardized nutritional risk screening tools have been developed, including the Controlling Nutritional Status (CONUT) score, the Prognostic Nutritional Index (PNI), and the Geriatric Nutritional Risk Index (GNRI) (17, 18). Higher CONUT scores, as well as lower PNI and GNRI scores, indicate a higher risk of malnutrition rather than a definitive diagnosis of malnutrition. Recent investigations have linked elevated preoperative nutritional risk, assessed via these tools, to an increased incidence of postoperative complications, including AKI (19, 20).

The present study aimed to systematically investigate the association between preoperative nutritional status, as evaluated by CONUT, GNRI, and PNI scores, and the risk of developing postoperative AKI in patients undergoing CABG. Beyond traditional regression analyses, we leveraged multiple machine learning algorithms to construct predictive models integrating nutritional indices with other clinical variables, thereby enabling more accurate and robust risk estimation. The best-performing model was externally validated in an independent cohort to assess its generalizability. To enhance interpretability, Shapley additive explanations (SHAP) were applied to quantify the contribution of each predictor to AKI risk. Finally, an interactive web-based tool was developed using the RShiny framework to facilitate individualized risk assessment and clinical application.

## 2 Methods

### 2.1 Data source

This retrospective analysis utilized data collected from two centers: the First Affiliated Hospital of Shandong First Medical University (June 2015 to October 2022) and the Affiliated Hospital of North Sichuan Medical College (January 2016 to January 2025). Data were extracted from electronic medical records, including demographic, laboratory, surgical, and postoperative outcome information, following standardized data collection protocols at both centers. Patients who underwent CABG were screened for eligibility. The inclusion criteria were: (1) patients aged 18 years or older; (2) patients who underwent isolated CABG surgery; and (3) availability of postoperative renal function data. The exclusion criteria were: (1) patients with chronic kidney disease (CKD) stage 5 or those undergoing maintenance dialysis prior to surgery; (2) patients who underwent combined major cardiac procedures, such as valve repair/replacement or aortic surgery; and (3) patients with severe infections, malignancies, or systemic diseases that could affect renal function, such as advanced liver failure, systemic autoimmune diseases (e.g., systemic lupus erythematosus), or hematological disorders. After applying these criteria, a total of 811 patients were included in the final analysis, comprising 714 from the Shandong cohort and 97 from the Sichuan cohort. A detailed flowchart of patient selection is provided in Supplementary Figure S1. This study was conducted in accordance with the TRIPOD guidelines for reporting clinical prediction models.

**Figure 1:**
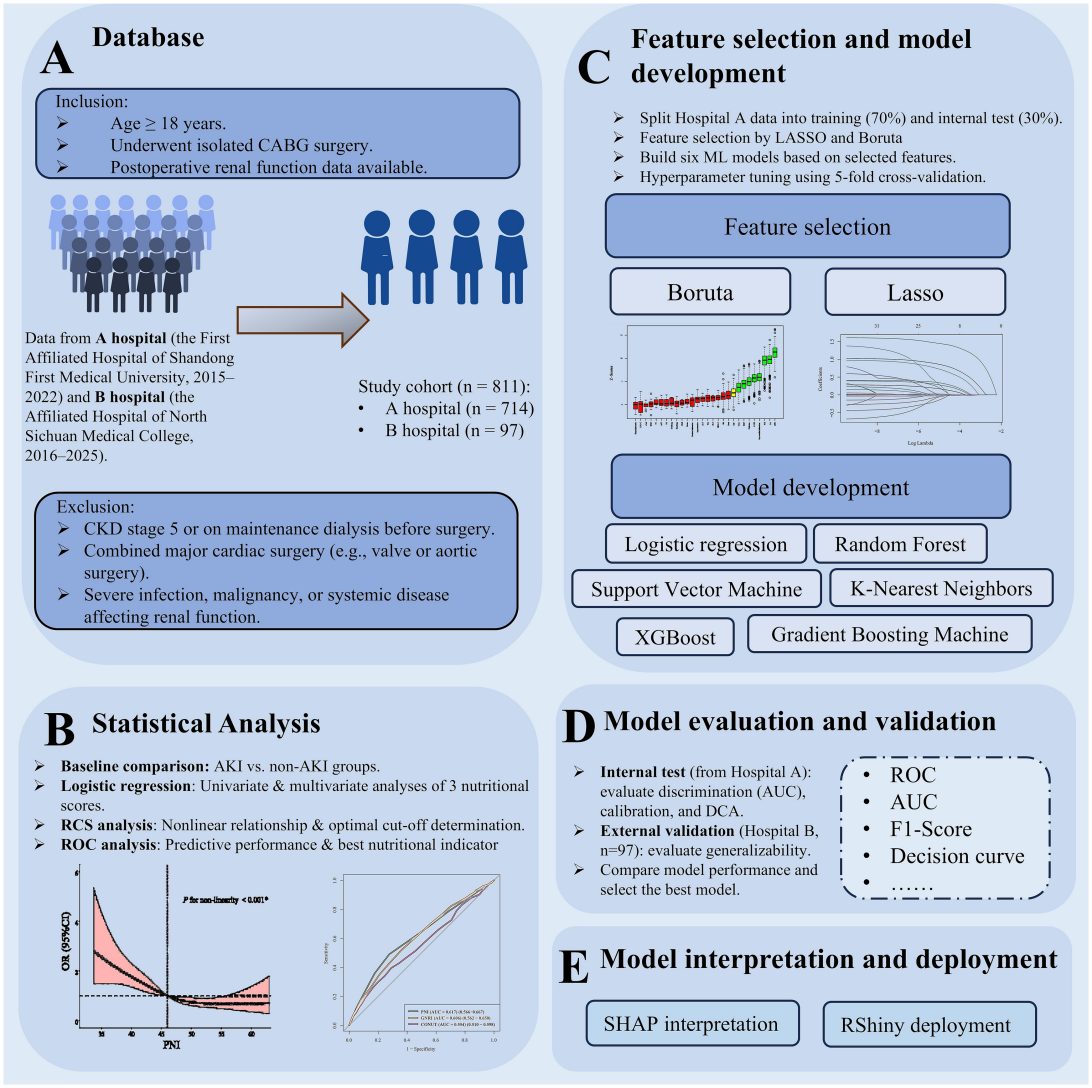
Workflow for predicting postoperative AKI after CABG using machine learning.**(A)** Patients from two hospitals were included/excluded, forming an 811-patient cohort. **(B)** Statistical analyses compared AKI/non-AKI groups and evaluated nutritional score performance. **(C)** Features were selected via Boruta/LASSO; six ML models were built and tuned. **(D)** Models were internally and externally validated to select the best performer. **(E)** SHAP interpretation and RShiny deployment completed the workflow.

A detailed summary of missing values for each variable, stratified by AKI status in the original dataset, is provided in Supplementary Table S1. Missing values were handled using multiple imputation with the “mice” package, with 5 imputations performed. A visual summary of missing data for patients without AKI and with AKI is shown in Supplementary Figure S2. The overall design and analytical workflow of this study are summarized in Figure 1.

### 2.2 Nutritional assessment

The preoperative nutritional status of patients was evaluated using three validated nutritional assessment tools: CONUT score, PNI, and GNRI. The CONUT score was calculated based on three laboratory parameters: serum albumin concentration, total lymphocyte count, and total cholesterol level (21). Each parameter was assigned a specific score according to predefined cut-off values, and the sum of the three scores yielded the total CONUT score, ranging from 0 to 12. The scoring system for CONUT was represented in Supplementary Table S2. Higher CONUT scores indicated poorer nutritional status. The PNI was determined using serum albumin concentration and total lymphocyte count (16), where lower values of either parameter indicated a worse nutritional status. A lower PNI score reflected a higher degree of malnutrition, and a score below 45 was considered indicative of nutritional risk. The GNRI was assessed using serum albumin concentration along with the ratio of actual body weight to ideal body weight (22). The ideal body weight was estimated based on an assumed body mass index (BMI) of 22 kg/m^2^ (12). If the actual body weight exceeded the ideal body weight, the ratio was set to 1. Higher GNRI scores indicated better nutritional status, while a GNRI score below 92 suggested moderate to severe malnutrition.

### 2.3 AKI assessment

The postoperative AKI was identified based on the creatinine criteria outlined by the Kidney Disease: Improving Global Outcomes (KDIGO) guidelines (23). Specifically, AKI was defined as an increase in serum creatinine by at least 26.5 μmol/L within 48 h or to 1.5 times or more the baseline value within 7 days after surgery. The baseline creatinine was determined as the most recent measurement obtained within 7 days before the operation.

### 2.4 Multivariable logistic regression and nonlinear association analysis

Three multivariate logistic regression models were constructed to evaluate the association between nutritional scores and AKI. Model 1 was unadjusted. Model 2 was adjusted for demographic and clinical variables, including age, sex, hypertension, hyperlipidemia, diabetes mellitus, prior percutaneous coronary intervention (PCI), and cardiopulmonary bypass (CPB). Model 3 was further adjusted for cardiac and biochemical variables, including left ventricular ejection fraction (LVEF), B-type natriuretic peptide (BNP), and uric acid (UA). Results are reported as odds ratio (OR) with 95% confidence interval (CI). To avoid multicollinearity, variables directly used in the calculation of nutritional scores (such as weight, height, BMI, lymphocyte count, serum albumin, and total cholesterol) were excluded from the regression models. The relationships between preoperative nutritional indices (treated as continuous variables) and AKI were further explored using restricted cubic spline (RCS) models to assess potential non-linear associations.

### 2.5 Feature selection

Prior to model development, receiver operating characteristic (ROC) analyses were performed for the three nutritional assessment tools (CONUT, PNI, and GNRI), and the one with the highest predictive performance was selected for inclusion. Feature selection was then conducted using the least absolute shrinkage and selection operator (LASSO) regression and the Boruta algorithm. Boruta, a random forest–based feature selection method, was implemented using the “Boruta” package in R (pValue = 0.01, maxRuns = 500) to identify variables significantly associated with the outcome. LASSO regression, performed with the “glmnet” package (family = “binomial,” alpha = 1), applied a penalty to shrink regression coefficients and enhance model robustness. The optimal penalty parameter (*λ*) was determined via 10-fold cross-validation using the cv.glmnet function. Finally, to ensure stable and interpretable predictors, only variables consistently identified by both Boruta and LASSO were retained for model construction.

### 2.6 Model development and validation

Based on the selected features and the optimal nutritional score, predictive models were constructed using six machine learning algorithms: logistic regression (LR), random forest (RF), k-nearest neighbors (KNN), support vector machine (SVM), gradient boosting machine (GBM), and extreme gradient boosting (XGB). PNI was entered as a continuous variable in all machine learning models. In logistic regression, non-linearity between PNI and AKI was modeled using restricted cubic splines based on prior RCS analysis. The Shandong cohort was randomly partitioned into training (70%) and internal validation (30%) subsets, while the Sichuan cohort served as an external validation set. All models were implemented using the Python package Scikit-learn (version 0.24.1).

Hyperparameter tuning was performed for each algorithm using grid search combined with 10 rounds of 10-fold cross-validation to identify the optimal settings that maximized predictive performance. The tuning process was conducted using the final feature subset selected by LASSO and Boruta (Supplementary Table S3).

Model performance was evaluated using multiple metrics, including accuracy, sensitivity, specificity, positive predictive value (PPV), negative predictive value (NPV), F1-score, kappa score, and the area under the receiver operating characteristic curve (AUC). In addition, decision curve analysis (DCA) was conducted to assess the potential clinical utility of each model in identifying patients at risk for AKI.

### 2.7 Model explanation and deployment

To enhance model interpretability, feature contributions were quantified using Shapley additive explanations (SHAP) (24), which provide both local (individual-level) and global (cohort-level) insights into the impact of each predictor on AKI risk. SHAP values were computed using the SHAP Python library (version 0.43.0). This analysis allowed visualization of feature importance, directionality, and interactions, thereby improving transparency and clinical interpretability of the predictive models. Based on the final selected model, an interactive web application was developed using RShiny, enabling real-time risk estimation for individual patients and facilitating potential integration into clinical decision-making workflows.

### 2.8 Statistical analysis

Continuous variables with a normal distribution are presented as mean ± standard deviation (SD) and compared using the independent samples Student’s t-test. Continuous variables with a non-normal distribution are presented as median (interquartile range, IQR) and compared using the Kruskal–Wallis test. Categorical variables are presented as counts (percentages) and compared using the Chi-square test. All statistical analyses were performed using R software (version 4.4.2; http://www.R-project.org), and a two-sided *p*-value <0.05 was considered statistically significant.

## 3 Result

### 3.1 Baseline characteristics of the patients

A total of 811 patients who underwent CABG were included in the analysis. The mean age was 63.82 ± 7.94 years, and 568 patients (70.0%) were male. Postoperative AKI occurred in 143 patients, yielding an incidence rate of 17.6% (Table 1). Compared with patients without AKI, those who developed AKI were more likely to have a history of hypertension, hyperlipidemia, diabetes mellitus, prior PCI, and to have undergone CPB. Biochemical assessments also showed that the AKI group had significantly elevated levels of BNP and UA, along with reduced LVEF and lower serum albumin levels (all *p* < 0.05). Nutritional status differed significantly between the two groups. Patients with AKI demonstrated poorer nutritional profiles, characterized by higher CONUT scores [2.00 (1.00–4.00) vs. 2.00 (1.00–3.00), *p* < 0.001], and lower GNRI [95.1 (88.3–100.2) vs. 97.2 (92.6–102.1), *p* < 0.001] and PNI values [43.2 (38.4–48.1) vs. 46.7 (42.8–50.4), *p* < 0.001].

**Table 1:** Baseline characteristics.

Characteristics	Overall (*n* = 811)	Non-AKI (*n* = 668)	AKI (*n* = 143)	*p* value
Age, years	63.82 (7.94)	63.76 (7.90)	64.13 (8.15)	0.570
Sex, %				0.726
Female	243 (30.0)	199 (29.8)	44 (30.8)	
Male	568 (70.0)	469 (70.2)	99 (69.2)	
Hypertension, %	503 (62.0)	400 (59.9)	103 (72.0)	0.003^*^
Hyperlipidemia, %	281 (34.6)	216 (32.3)	65 (45.5)	0.004^*^
Diabetes, %	113 (13.9)	79 (11.8)	34 (23.8)	0.002^*^
Myocardial infarction, %	28 (3.5)	22 (3.3)	6 (4.2)	0.543
Cerebral infarction, %	61 (7.5)	51 (7.6)	10 (7.0)	0.778
PCI, %	118 (14.6)	66 (9.9)	52 (36.4)	<0.001^*^
Smoking, %	400 (49.3)	331 (49.6)	69 (48.3)	0.764
Drinking, %	336 (41.4)	275 (41.2)	61 (42.7)	0.728
CPB, %	271 (27.3)	164 (24.6)	57 (39.9)	<0.001^*^
LVEF, %	57.16 (9.27)	57.77 (9.32)	54.36 (8.55)	<0.001^*^
BMI, kg/m^2^	25.45 (3.38)	25.40 (3.40)	25.00 (3.31)	0.371
WBC, × 10^9^/L	6.38 (1.67)	6.35 (1.64)	6.53 (1.80)	0.181
Lymph, × 10^9^/L	1.76 (0.58)	1.76 (0.56)	1.75 (0.68)	0.872
Mono, × 10^9^/L	0.52 (0.18)	0.52 (0.18)	0.54 (0.18)	0.147
Neut, × 10^9^/L	3.89 (1.38)	3.86 (1.34)	4.04 (1.53)	0.126
Hb, g/L	131.72 (16.88)	131.87 (16.43)	131.03 (18.88)	0.551
PLT, × 10^9^/L	223.45 (60.47)	225.07 (61.03)	215.92 (57.36)	0.068
cTnI, ng/mL	0.59 (2.80)	0.60 (2.89)	0.55 (2.34)	0.84
BNP, pg./mL	265.88 (454.15)	251.24 (438.28)	334.18 (517.82)	0.027^*^
ALT, U/L	31.47 (33.06)	32.14 (34.60)	28.32 (24.42)	0.163
AST, U/L	28.52 (33.04)	28.37 (30.29)	29.23 (43.73)	0.756
ALB, g/L	40.35 (3.83)	40.41 (3.79)	39.06 (4.00)	<0.001^*^
Urea (mmol/L)	5.78 (2.59)	5.71 (2.59)	6.12 (2.58)	0.057
UA, μmol/L	315.69 (94.49)	311.52 (94.96)	335.16 (90.00)	0.002^*^
TC, mmol/L	3.96 (1.07)	3.99 (1.08)	3.84 (0.98)	0.084
TG (mmol/L)	1.49 (0.91)	1.50 (0.92)	1.47 (0.84)	0.673
HDL-C (mmol/L)	1.01 (0.23)	1.01 (0.23)	1.00 (0.25)	0.783
LDL-C (mmol/L)	2.17 (0.91)	2.18 (0.92)	2.13 (0.84)	0.478
CONUT	2.00 [1.00, 4.00]	2.00 [1.00–3.00]	2.00 [1.00–400]	<0.001^*^
GNRI	96.6 [91.5, 101.2]	97.2 [92.6, 102.1]	95.1 [88.3, 100.2]	<0.001^*^
PNI	45.5 [41.3, 50.1]	46.7 [42.8, 50.4]	43.2 [38.4, 48.1]	<0.001^*^

Data are mean (standard deviation), median [interquartile range], or *n* (%). AKI indicates acute kidney injury; PCI, percutaneous coronary intervention; CPB, cardiopulmonary bypass; LVEF, left ventricular ejection fraction; BMI, body mass index; WBC, white blood cell count; Lymph, lymphocyte count; Mono, monocyte count; Neut, neutrophil count; Hb, hemoglobin; PLT, platelet count; cTnI, cardiac troponin I; BNP, B-type natriuretic peptide; ALT, alanine aminotransferase; AST, aspartate aminotransferase; ALB, albumin; Urea, urea; UA, uric acid; TC, total cholesterol; TG, triglyceride; HDL-C, high-density lipoprotein cholesterol; LDL-C, low-density lipoprotein cholesterol; CONUT, controlling nutritional status; GNRI, geriatric nutritional risk index; PNI, prognostic nutritional index. *p < 0.05.

### 3.2 The association between nutritional risk and AKI

**Figure 2:**
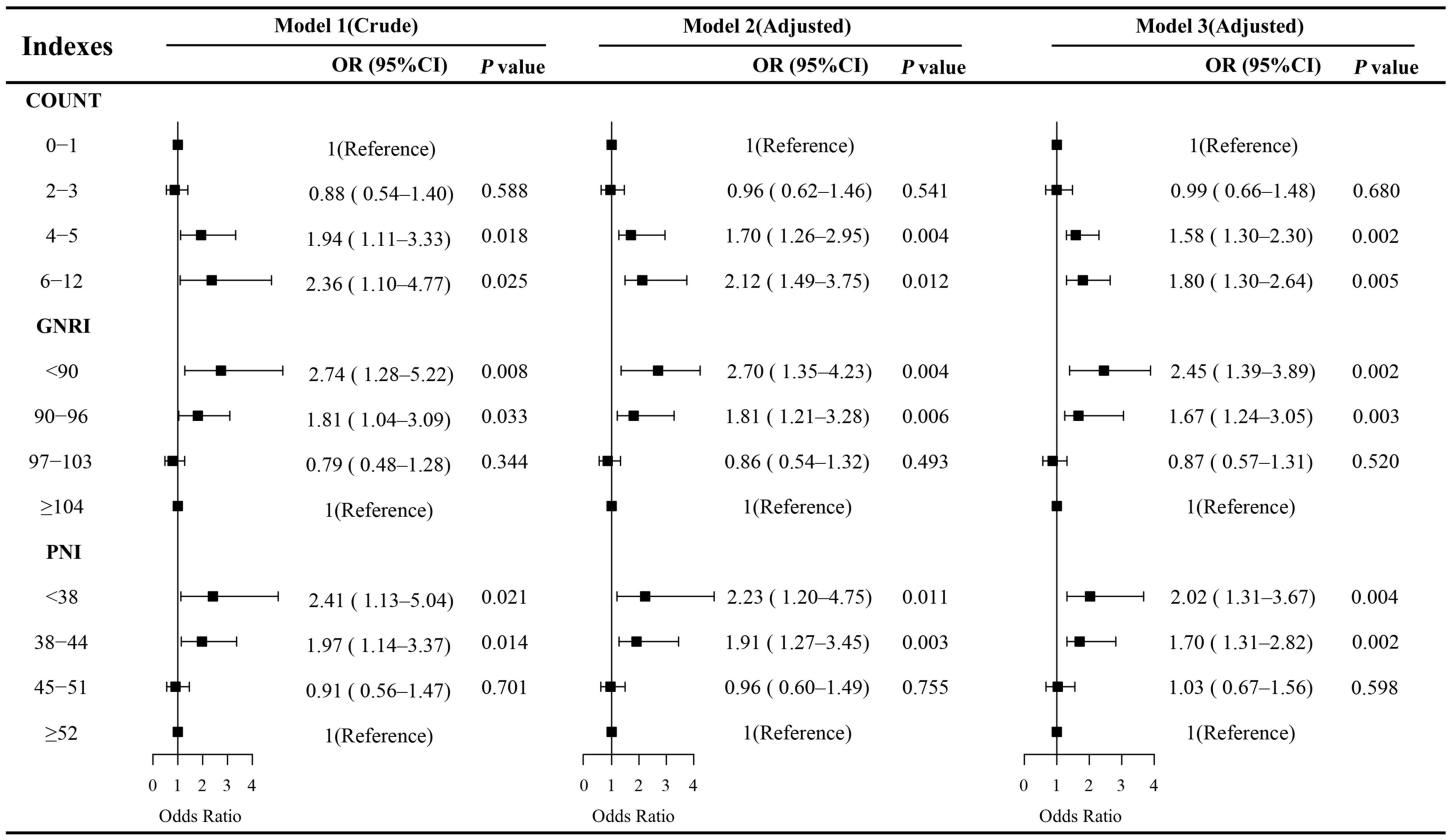
Univariate and multivariate logistics analyses of the association between 3 malnutrition indexes and AKI. Model 1 was unadjusted. Model 2 was adjusted for demographic and clinical variables, including age, sex, hypertension, hyperlipidemia, diabetes mellitus, prior percutaneous coronary intervention (PCI), and cardiopulmonary bypass (CPB). Model 3 was additionally adjusted for cardiac function and biochemical variables, including left ventricular ejection fraction (LVEF), B-type natriuretic peptide (BNP), and uric acid (UA).

The association between preoperative nutritional status and the risk of AKI following CABG was examined using univariate and multivariate logistic regression analyses (Figure 2). Nutritional scores were categorized based on established cut-off values, with the lowest nutritional risk groups set as reference (CONUT: 0–1; PNI: ≥52; GNRI: ≥104). In univariate analysis, poor nutritional status was significantly associated with an increased risk of AKI, including CONUT (6–12 vs. 0–1: OR = 2.36, 95% CI: 1.10–4.77, *p* = 0.025), GNRI (<90 vs. ≥104: OR = 2.74, 95% CI: 1.28–5.22, *p* = 0.008), and PNI (<38 vs. ≥52: OR = 2.41, 95% CI: 1.13–5.04, *p* = 0.021). In multivariate logistic regression analyses, the association between malnutrition and AKI remained statistically significant across both Model 1 and Model 2, regardless of the specific nutritional index applied, suggesting that malnutrition serves as an independent risk factor for AKI.

**Figure 3:**
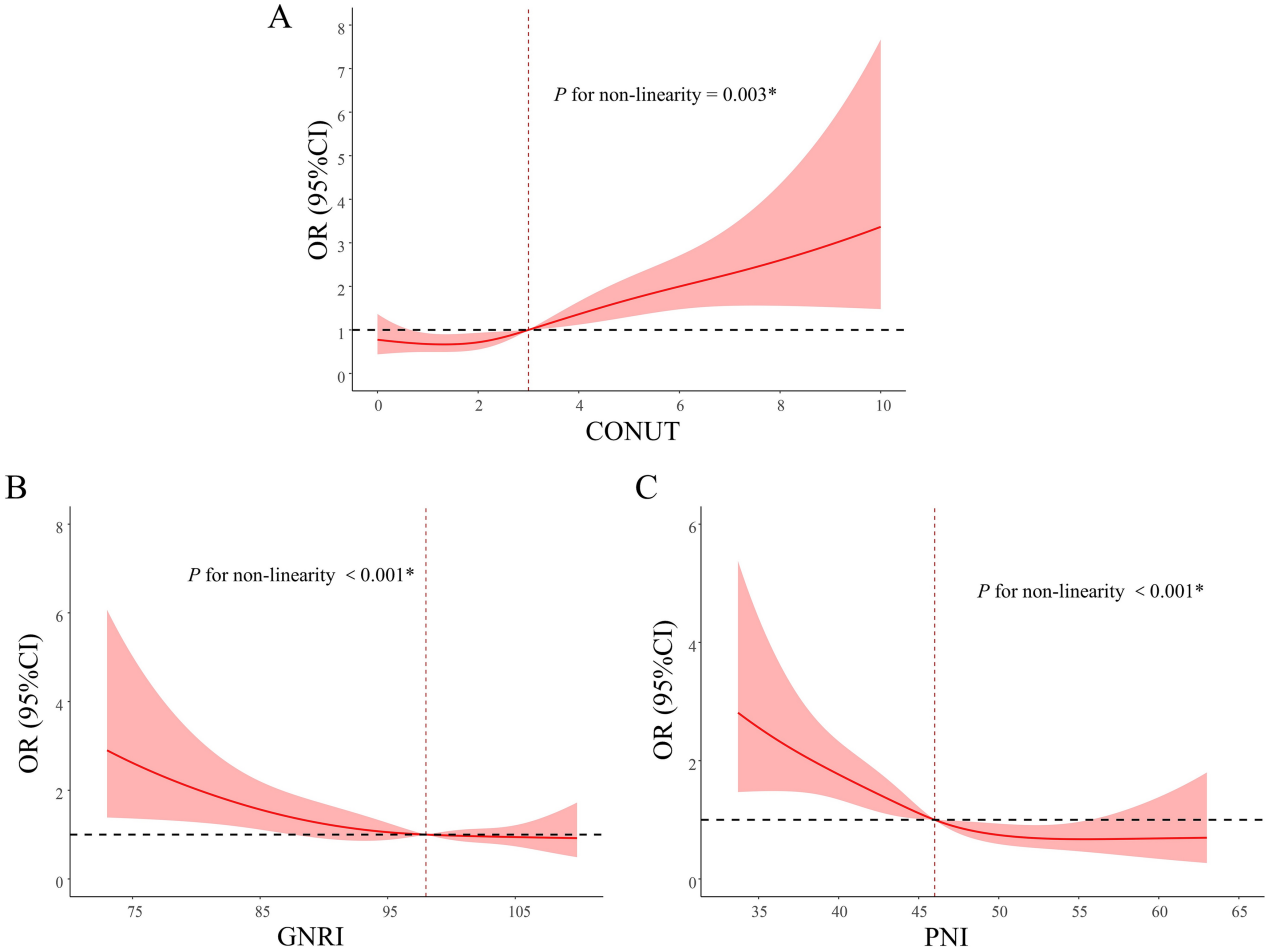
Restricted cubic spline analyses of the association between nutritional scores and the risk of AKI. The plots illustrate the dose–response relationship between nutritional scores and postoperative AKI using restricted cubic spline models: **(A)** CONUT, **(B)** GNRI, and **(C)** PNI. The solid lines represent the estimated odds ratios, and the shaded areas indicate the 95% confidence intervals. AKI, acute kidney injury; CI, confidence interval; CONUT, Controlling Nutritional Status; GNRI, Geriatric Nutritional Risk Index; PNI, Prognostic Nutritional Index. *p < 0.05.

**Figure 4:**
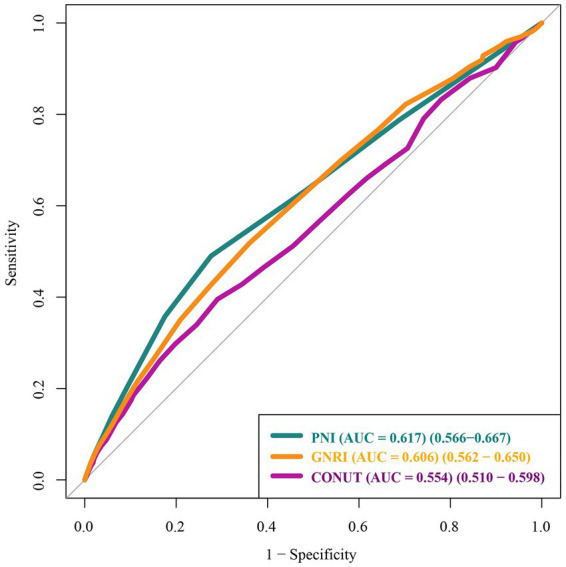
Receiver operating characteristic (ROC) curves of nutritional scores for predicting AKI. CONUT, controlling nutritional status; GNRI, geriatric nutritional risk index; PNI, prognostic nutritional index.

To further explore the dose–response relationship, RCS analysis was conducted with each nutritional score as a continuous variable (Figure 3). RCS analyses revealed nonlinear associations between each nutritional score and AKI risk (all *P* for non-linearity < 0.05). The inflection points were observed around a CONUT score of 3, a GNRI of 98, and a PNI of 46, respectively. ROC curve analyses demonstrated that all three nutritional scores had modest predictive performance for AKI, with AUC values of 0.554 (95% CI: 0.510–0.598) for CONUT, 0.606 (95% CI: 0.562–0.650) for GNRI, and 0.617 (95% CI: 0.566–0.667) for PNI, suggesting that PNI may have greater clinical utility in identifying patients at risk of postoperative AKI (Figure 4).

**Figure 5:**
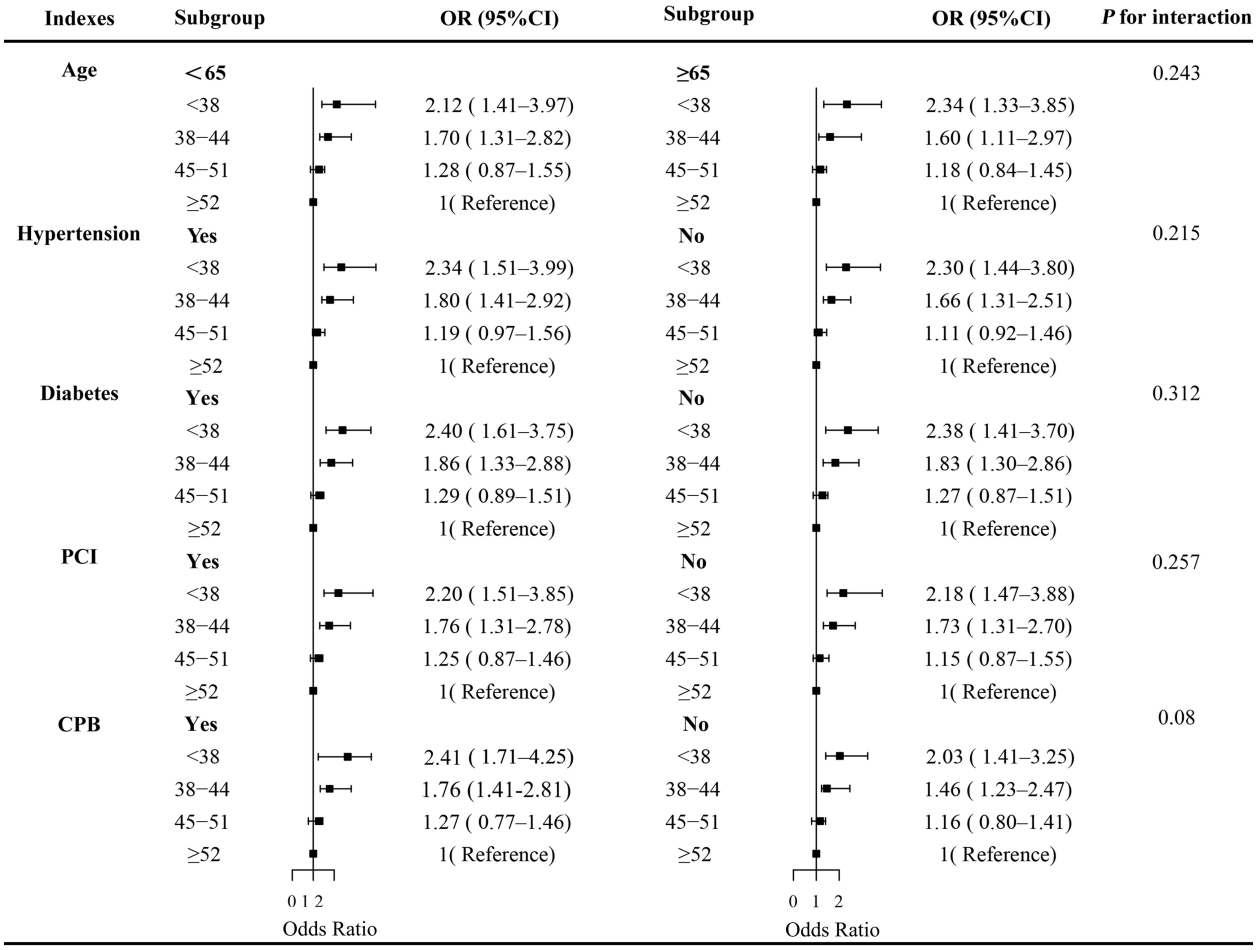
Forest plot of stratified analyses evaluating the association between PNI and postoperative AKI across predefined subgroups. Odds ratios (dots) and 95% confidence intervals (horizontal lines) were derived from multivariate logistic regression models adjusted for relevant covariates. *p*-values for interaction are shown for each subgroup. AKI, acute kidney injury; PNI, prognostic nutritional index; PCI, percutaneous coronary intervention; CPB, cardiopulmonary bypass.

Stratified analyses were conducted to examine whether the association between PNI and postoperative AKI differed across key demographic and clinical subgroups. In the fully adjusted model (Figure 5), the inverse relationship between PNI and AKI risk remained broadly consistent across all examined subgroups, including age (< 65 vs. ≥ 65 years), hypertension, prior PCI, diabetes, and history of CPB, with no significant effect modification observed.

### 3.3 Feature selection

**Figure 6:**
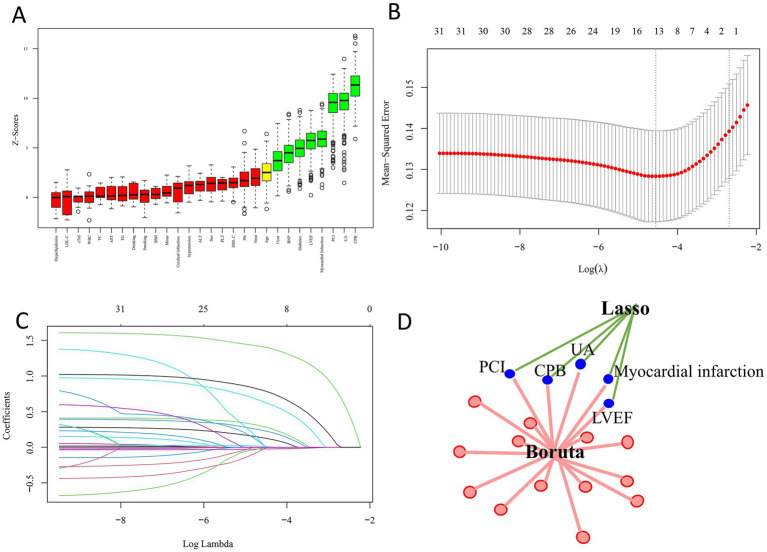
Feature selection results. **(A)** Feature importance ranking identified by the Boruta algorithm. **(B)** LASSO regression with optimal lambda values (*λ*.min and λ.1se) indicated by dashed lines. **(C)** Coefficient trajectories of candidate variables across the LASSO regularization path. **(D)** Common predictors selected by both Boruta and LASSO, including CPB, UA, PCI, myocardial infarction, and LVEF.

To further refine the predictive model, feature selection was performed using both the Boruta algorithm and LASSO regression. The Boruta algorithm, an extension of the random forest method that evaluates the importance of each variable against randomly permuted shadow features, identified eight key predictors: CPB, UA, PCI, myocardial infarction (MI) history, LVEF, diabetes mellitus, BNP, and urea (Figure 6A). In parallel, LASSO regression, a penalized shrinkage method that simultaneously conducts variable selection and regularization, identified the following predictors: CPB, UA, smoking status, drinking status, hypertension, PCI, MI history, and LVEF (Figures 6B,C).

By comparing the outputs of both methods, we determined the intersection of variables consistently selected by Boruta and LASSO. This common subset—CPB, UA, PCI, MI, and LVEF—was retained as the core feature set for model construction (Figure 6D). Considering prior evidence supporting the role of nutritional status in AKI and the relatively better performance of PNI compared with CONUT and GNRI in our cohort, PNI was also incorporated into the final model.

### 3.4 Model performance

**Figure 7:**
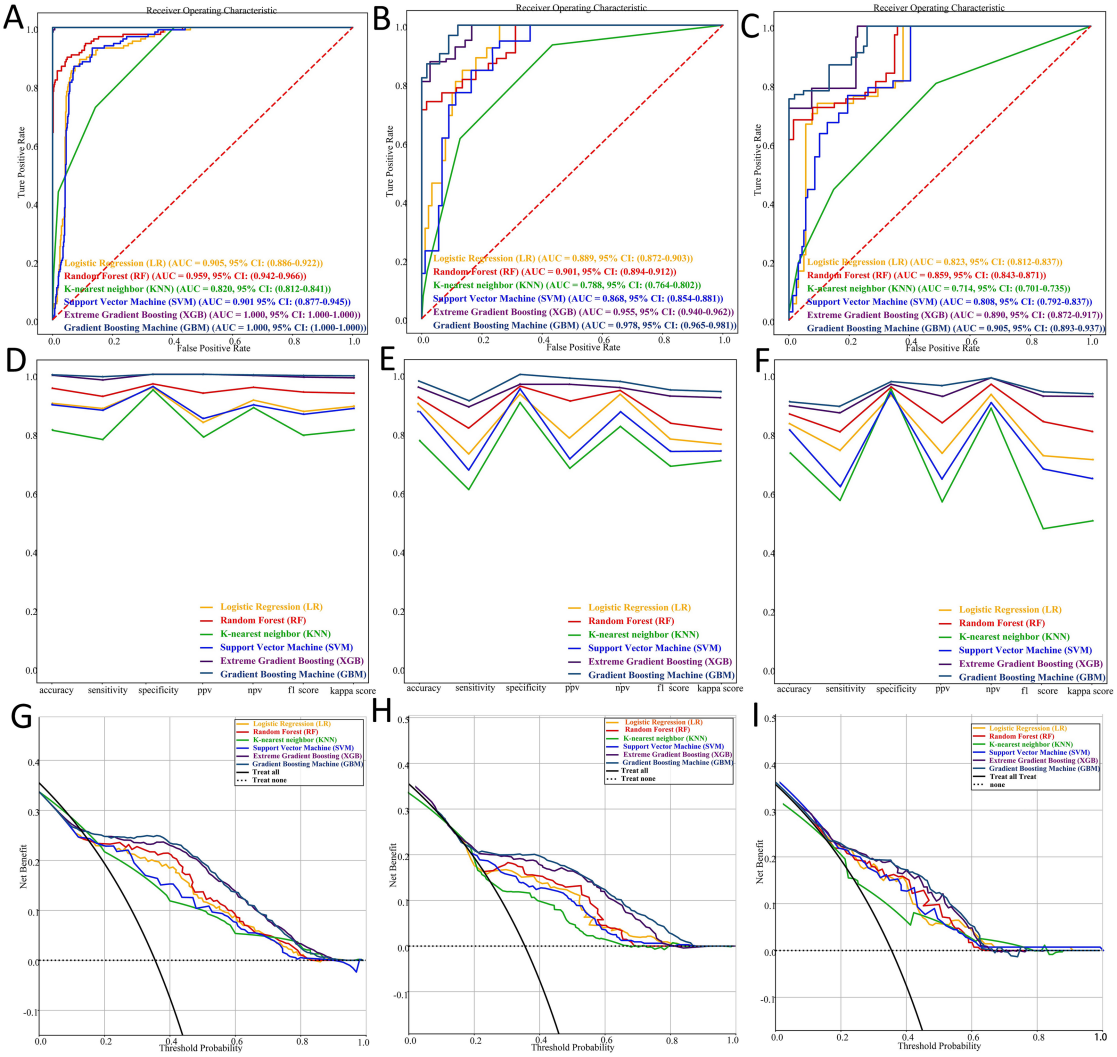
Performance and comparison of six predictive models. **(A–C)** Receiver operating characteristic (ROC) curves for the training set, internal validation set, and external validation set. **(D–F)** Comprehensive model performance metrics—including accuracy, sensitivity, specificity, positive predictive value (PPV), negative predictive value (NPV), F1-score, and kappa coefficient—for the training, internal validation, and external validation datasets. **(G–I)** Decision curve analysis (DCA) demonstrating the net clinical benefit of each model across a range of threshold probabilities in the training, internal validation, and external validation cohorts.

After ten rounds of 10-fold cross-validation, six machine learning models were developed and evaluated. In the training dataset, the models achieved the following AUC values: GBM 1.000 (95% CI: 1.000–1.000), XGB 1.000 (95% CI: 1.000–1.000), RF 0.959 (95% CI: 0.942–0.966), LR 0.905 (95% CI: 0.886–0.922), SVM 0.901 (95% CI: 0.877–0.945), and KNN 0.820 (95% CI: 0.812–0.841) (Figure 7A). In the internal validation cohort, the GBM model demonstrated the highest discriminatory ability with an AUC of 0.978 (95% CI: 0.965–0.981) (Figure 7B). Consistent with these findings, the GBM model also showed the best predictive performance in the external validation cohort, yielding an AUC of 0.905 (95% CI: 0.893–0.937) (Figure 7C). Comprehensive performance metrics—including accuracy, sensitivity, specificity, PPV, NPV, F1-score, and kappa—were calculated for all models in the training, internal validation, and external validation sets (Figures 7D–F). Decision curve analysis (DCA) further demonstrated that the GBM model provided the greatest net clinical benefit across the full threshold probability range (0–1.0) in all three datasets, followed by the XGB model (Figures 7G–I).

### 3.5 SHAP interpretation and web deployment

**Figure 8:**
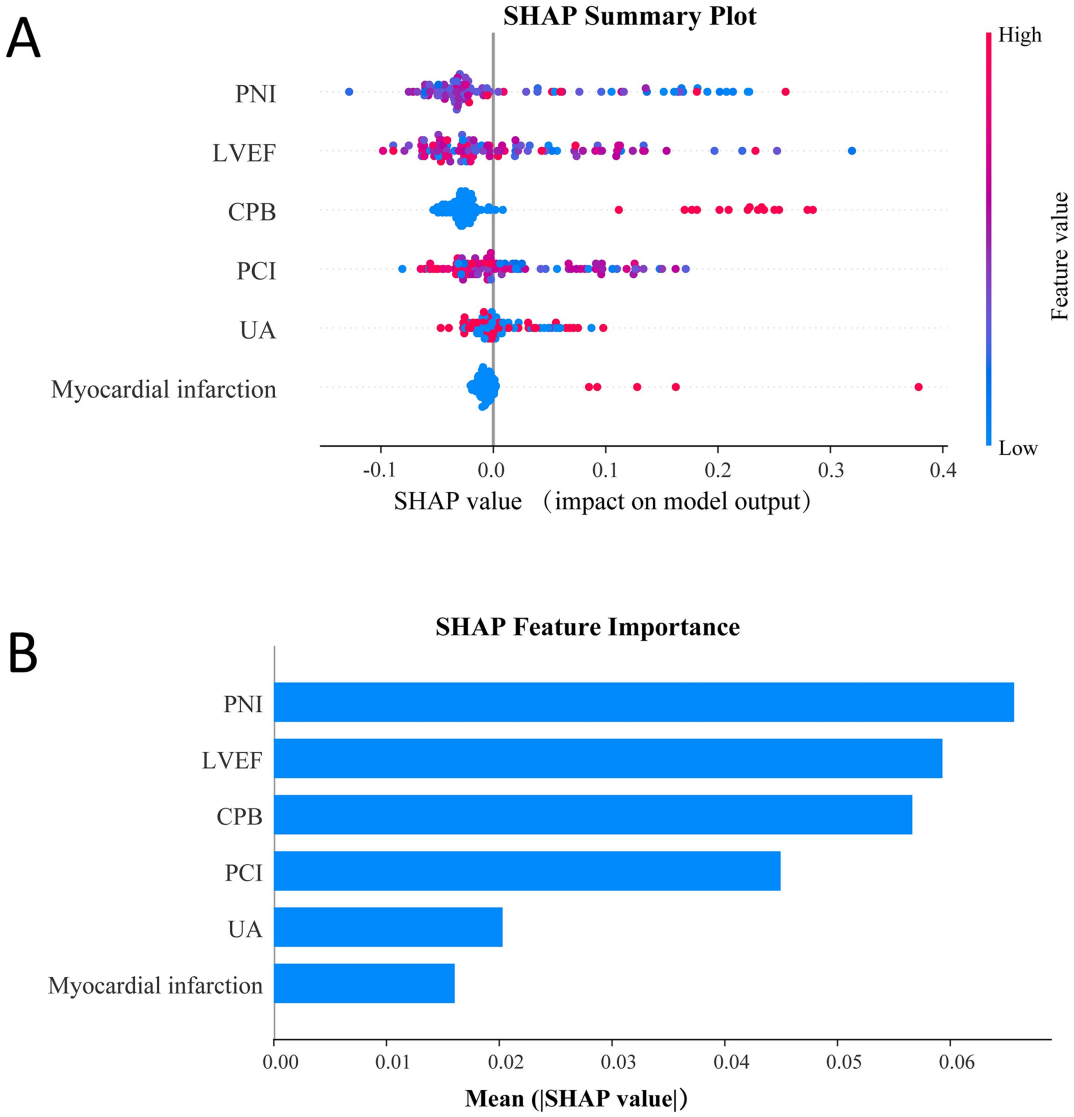
SHAP interpretability of the GBM model. **(A)** SHAP summary plot showing feature contributions to model predictions. **(B)** Mean absolute SHAP values ranking feature importance.

We employed SHAP to interpret the output of the final GBM model by quantifying the contribution of each predictor to the estimated AKI risk. In Figure 8A, the SHAP summary plot illustrates the impact of individual features, where each point represents a single patient, and the color gradient from blue (low feature values) to red (high feature values) reflects the corresponding feature magnitude. The vertical axis ranks predictors by importance, demonstrating how variations in feature values influence their SHAP contributions. Figure 8B displays the mean absolute SHAP values for all predictors, providing an aggregated measure of global feature importance. The results highlight PNI, LVEF, and CPB as the top three contributors to model predictions, underscoring their substantial influence on postoperative AKI risk.

**Figure 9:**
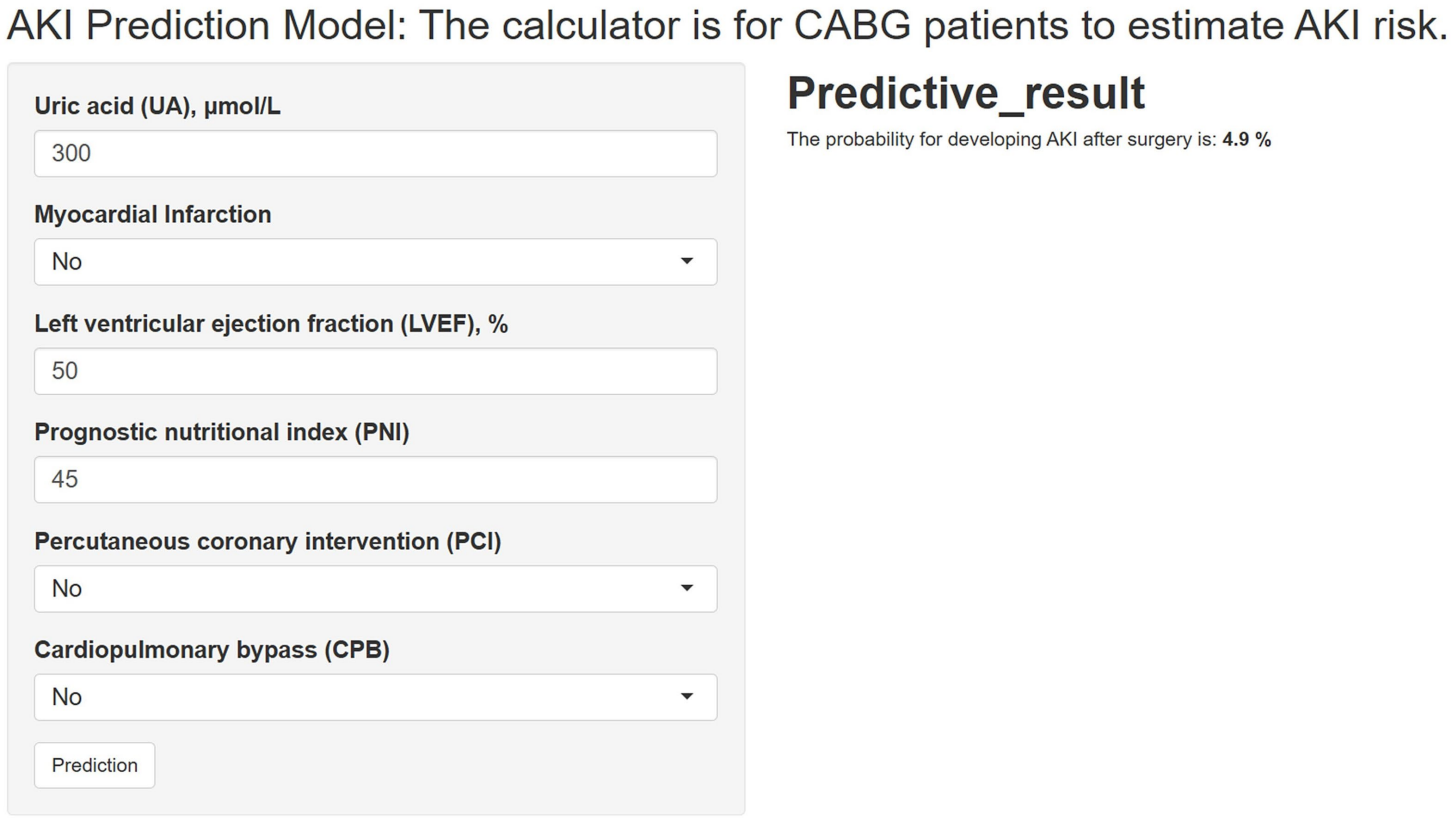
Web-based calculator for predicting postoperative AKI risk in CABG patients using the GBM model.

To enhance clinical applicability, the final GBM model was deployed as an interactive web-based tool (Figure 9). By entering values for the selected predictors, clinicians can obtain an individualized AKI risk estimate in real time. The application is accessible at: https://dpn-prediction.shinyapps.io/0702AKImodel/.

## 4 Discussion

In this retrospective study of 811 patients undergoing CABG, we first examined the association between preoperative nutritional status—evaluated using CONUT, PNI, and GNRI—and the risk of postoperative AKI. All three indices demonstrated that poorer nutritional status was significantly correlated with higher AKI incidence, and these associations remained robust after adjustment for demographic factors, comorbidities, surgical variables, and key laboratory parameters. Based on the best-performing nutritional score, we then applied LASSO and Boruta algorithms to identify additional predictors and developed multiple machine learning models for AKI risk estimation. The selected model demonstrated strong discrimination, high accuracy, and favorable clinical utility across the training, internal validation, and external validation cohorts. Finally, model interpretability was enhanced through SHAP analysis, and a web-based calculator was deployed to facilitate clinical application.

Malnutrition is increasingly recognized as a major contributor to adverse outcomes across a wide range of diseases, including cardiovascular disease (25), chronic kidney disease (26), cancer (27), and stroke (28). Traditional nutritional screening tools—such as MNA-SF (29), MUST (30), and NRS-2002 (31)—are widely recommended but rely on subjective assessments that may be influenced by recall bias or communication difficulties, particularly in acutely ill or elderly patients. In contrast, the GNRI, PNI, and CONUT scores provide objective, laboratory-based evaluations derived from routine clinical parameters, offering a more standardized and reproducible approach to assessing nutritional risk.

In our study, all three nutritional indices were significantly associated with postoperative AKI, consistent with the biological plausibility linking malnutrition to renal vulnerability. Malnutrition may impair immune function (32), intensify systemic inflammation (33), reduce renal perfusion reserve (34), and worsen endothelial dysfunction (35), thereby increasing susceptibility to kidney injury during cardiac surgery. Although prior studies in non-cardiac surgical populations have reported similar associations (36, 37), evidence in CABG patients remains limited. A recent MIMIC-IV analysis identified malnutrition as a predictor of AKI among elderly CABG patients (38), and another single-center study showed that lower PNI independently predicted AKI (39). Our work extends these findings by examining a broader CABG population without age restrictions and directly comparing three objective nutritional indices, providing more comprehensive evidence for the prognostic relevance of preoperative nutritional status.

The three nutritional indices evaluated in this study (GNRI, PNI and CONUT) are objective, laboratory-based measures, yet they differ in their components, target populations and clinical applicability. PNI, calculated from serum albumin and lymphocyte count, provides a straightforward assessment of nutritional and immune status and is easily implemented in routine practice. CONUT incorporates albumin, lymphocyte count and total cholesterol, although its accuracy may be compromised in patients receiving lipid-lowering therapy, where cholesterol levels decrease independently of nutritional state and may lead to misclassification. GNRI, originally developed for older adults (38), combines albumin with the ratio of actual to ideal body weight and is particularly useful in elderly or chronically ill individuals, although body-weight–related parameters can be influenced by fluid retention or sarcopenia. In our cohort, all three indices were independently associated with postoperative AKI, and PNI demonstrated the highest predictive value with an AUC of 0.617. Because of its superior performance, PNI was integrated with key clinical predictors identified through Boruta and LASSO, including PCI, CPB duration, MI history, LVEF and UA, to construct six machine-learning models. To avoid multicollinearity, the individual components of PNI were not reintroduced into the multivariable analyses. Although the training set showed an AUC of 1.0, cross-validation, regularization, feature selection, and external validation were applied to minimize overfitting. The overall findings support the relevance of nutritional assessment and its combination with clinical variables for improving postoperative AKI risk stratification in patients undergoing CABG.

Compared with existing AKI prediction models after CABG, our GBM model demonstrated stronger discriminatory ability and clinical practicality. Prior studies reported AUCs around 0.73–0.78 (40, 41), but these models included many variables, 26 in one study and 11 in another, which may introduce redundancy and noise and reduce predictive accuracy. In contrast, our approach minimized variable redundancy and enhanced model robustness by selecting key predictors via Boruta and LASSO. Additionally, the inclusion of nutritional risk via PNI captures a clinically relevant factor, as malnutrition may predispose patients to AKI through impaired renal perfusion, oxidative stress, and immune dysfunction. Our GBM model achieved an AUC of 0.905 in the external validation cohort, indicating superior predictive accuracy. In addition, the incorporation of SHAP interpretability and deployment as a web-based calculator further enhances transparency and clinical usability. These advantages suggest that our model provides a more reliable and accessible tool for AKI risk assessment after CABG.

This study has several limitations. First, the retrospective and single-center design may introduce selection bias and limit the generalizability of the findings. Although an independent cohort was used for external validation, the sample size was relatively small, and larger multicenter or prospective studies are needed to further verify the reliability of the results. Second, only three objective nutritional indices (CONUT, GNRI, and PNI) were evaluated, while commonly used subjective screening tools such as NRS-2002 and MUST were not included. Incorporating both objective and subjective assessments may provide a more comprehensive evaluation of nutritional status. Future studies incorporating comprehensive malnutrition criteria, including inflammation, nutrient intake and assimilation, and muscle mass, may further enhance postoperative AKI risk prediction after cardiac surgery. Data on fasting blood glucose, HbA1c, and perioperative blood pressure were not available, which precluded assessment of glycemic or blood pressure control on AKI risk. Third, several important perioperative parameters, including fluid balance, transfusion volume, intraoperative hemodynamics, vasopressor use, nephrotoxic exposures, and contrast timing, were not available and therefore not included in the analysis, which may influence the accuracy of risk estimation. Finally, AKI was defined solely by serum creatinine according to KDIGO criteria. The lack of urine output data may have led to underestimation or misclassification of postoperative AKI.

## 5 Conclusion

In this study, preoperative nutritional status assessed by CONUT, GNRI, and PNI was independently associated with the risk of postoperative AKI in patients undergoing CABG, with PNI demonstrating the strongest predictive value. By integrating PNI with key clinical variables, we developed multiple machine-learning models, among which the GBM model achieved the highest discrimination and consistent performance across internal and external validation. SHAP analysis further enhanced model interpretability, and the web-based calculator provides a practical tool to support individualized AKI risk assessment. These findings highlight the clinical importance of nutritional evaluation before CABG and underscore the potential of combining objective nutritional indices with advanced modeling techniques to improve early risk stratification and guide perioperative management.

## Data Availability

The raw data supporting the conclusions of this article will be made available by the authors, without undue reservation.
