# Supplementary material for: Development and external validation of a machine learning model based on preoperative nutritional status for predicting acute kidney injury after coronary artery bypass grafting
Source: Front Nutr. 2026 Mar 13;13:1750814. doi: 10.3389/fnut.2026.1750814 (PMC13023136; doi:10.3389/fnut.2026.1750814)
Supplement: Supplementary file 1 [file Table_1.DOCX]

Supplementary Material


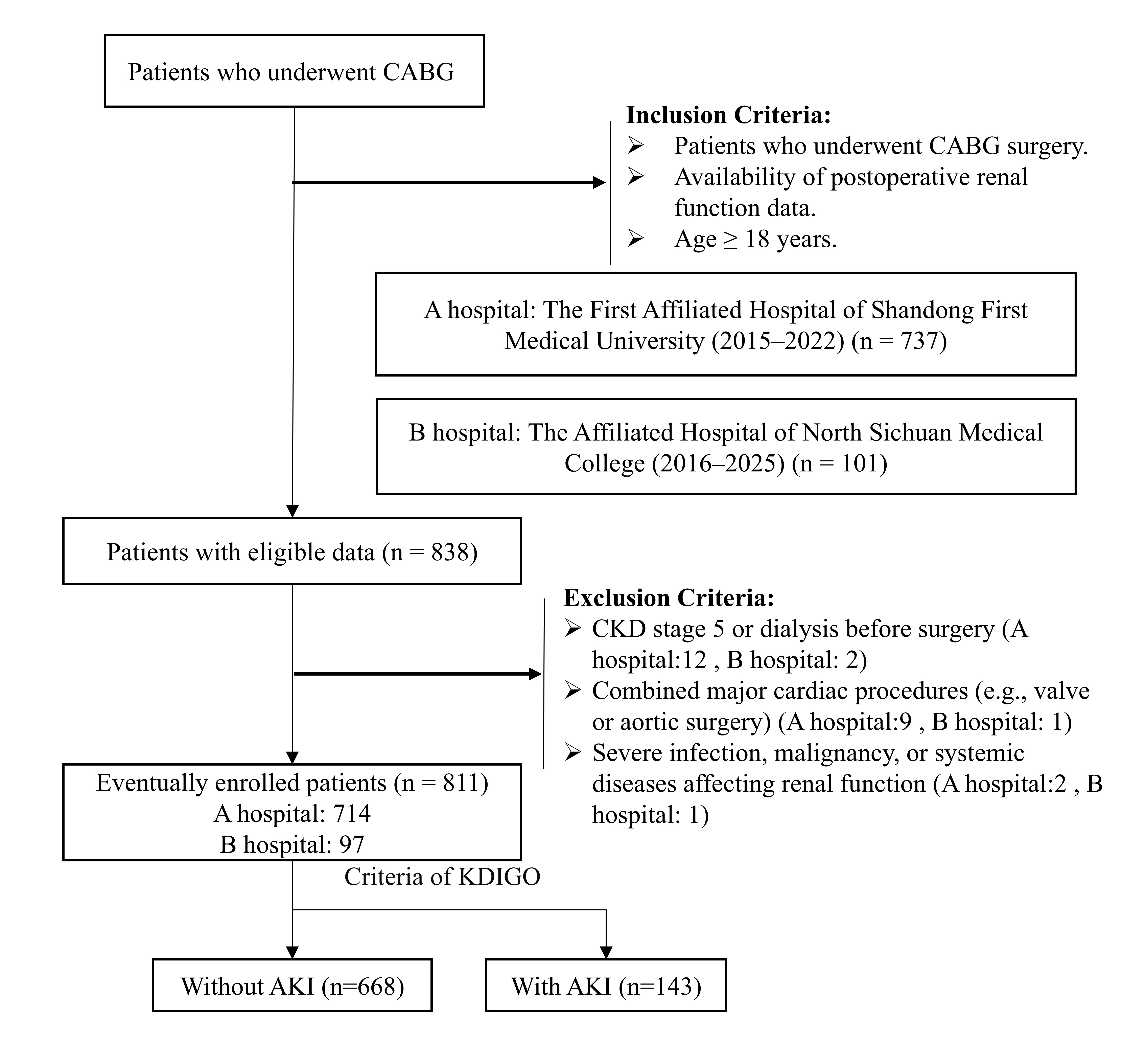


**Supplementary Figure 1.** Patient selection flowchart showing exclusions and final cohort.


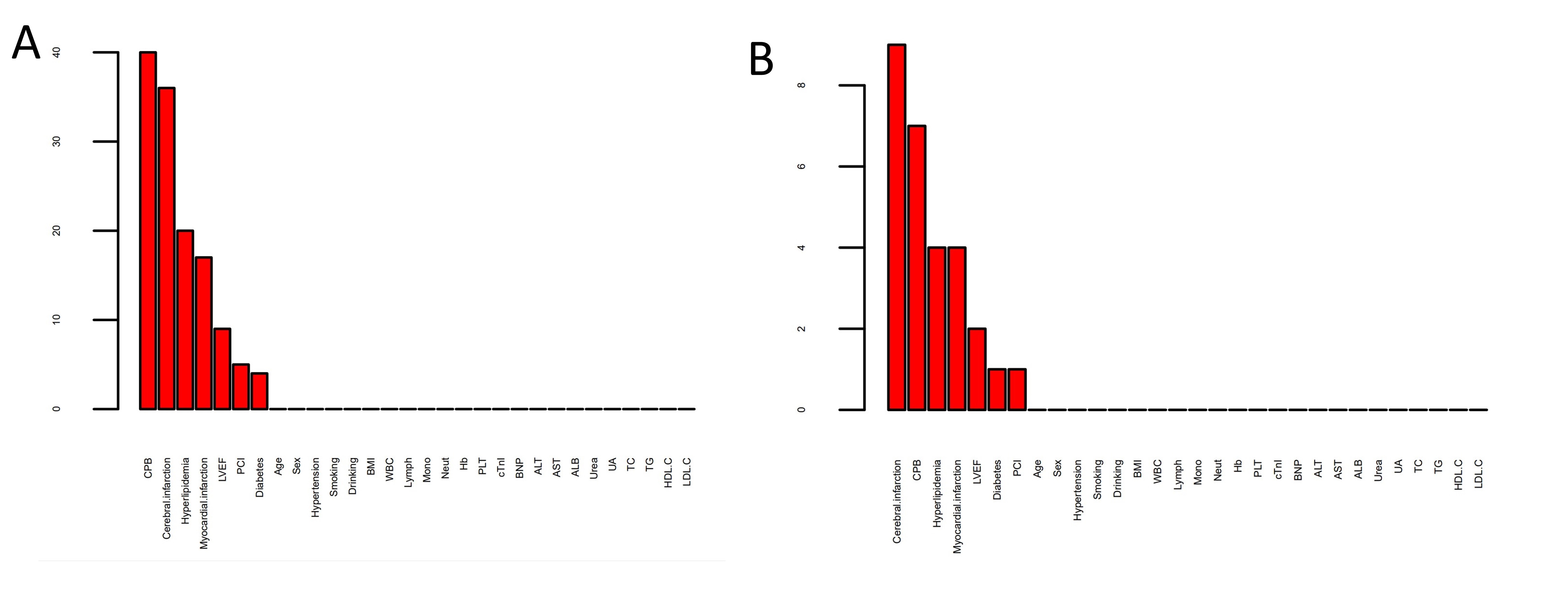


**Supplementary Figure 2.** Missing data patterns for all variables. Panel A: No-AKI; Panel B: AKI.

**Table S1. Missing data summary for all variables stratified by AKI status**

| Variable | Non-AKI group n (%) missing | AKI group n (%) missing | Total n (%) missing | Imputation applied |
| --- | --- | --- | --- | --- |
| Age | 0 | 0 | 0 | No |
| Sex | 0 | 0 | 0 | No |
| Hypertension | 0 | 0 | 0 | No |
| Hyperlipidemia | 20 (2.99) | 4 (2.80) | 24 (2.96) | Yes |
| Diabetes | 4 (0.60) | 1 (0.70) | 5 (0.62) | Yes |
| Myocardial infarction | 17 (2.54) | 4 (2.80) | 25 (2.59) | Yes |
| Cerebral infarction | 36 (5.39) | 9 (6.29) | 45 (5.55) | Yes |
| PCI | 5 (0.75) | 1(0.70) | 6 (0.74) | Yes |
| Smoking | 0 | 0 | 0 | No |
| Drinking | 0 | 0 | 0 | No |
| CPB | 40 (5.99) | 7 (4.90) | 47 (5.80) | Yes |
| LVEF | 9 (1.35) | 2 (1.40) | 11 (1.43) | Yes |
| BMI | 0 | 0 | 0 | No |
| WBC | 0 | 0 | 0 | No |
| Lymph | 0 | 0 | 0 | No |
| Mono | 0 | 0 | 0 | No |
| Neut | 0 | 0 | 0 | No |
| Hb | 0 | 0 | 0 | No |
| PLT | 0 | 0 | 0 | No |
| cTnI | 0 | 0 | 0 | No |
| BNP | 0 | 0 | 0 | No |
| ALT | 0 | 0 | 0 | No |
| AST | 0 | 0 | 0 | No |
| ALB | 0 | 0 | 0 | No |
| Urea | 0 | 0 | 0 | No |
| UA | 0 | 0 | 0 | No |
| TC | 0 | 0 | 0 | No |
| TG | 0 | 0 | 0 | No |
| HDL-C | 0 | 0 | 0 | No |
| LDL-C | 0 | 0 | 0 | No |

**Table S2. Scoring system for the CONUT**

| Parameter | None | Light | Moderate | Severe |
| --- | --- | --- | --- | --- |
| Serum albumin (g/dL) | ≥ 3.50 | 3.00-3.49 | 2.50-2.99 | < 2.50 |
| Score | 0 | 2 | 4 | 6 |
| Total lymphocyte count (/mm^3^) | ≥ 1600 | 1200-1599 | 800-1199 | < 800 |
| Score | 0 | 1 | 2 | 3 |
| Total cholesterol (mg/dL) | ≥ 180 | 140-179 | 100-139 | < 100 |
| Score | 0 | 1 | 2 | 3 |

**Table S3. Hyperparameters for nine models**

| Model | Hyperparameter | Candidate Hyperparameter Ranges |
| --- | --- | --- |
| Logistic Regression (LR) | none | none |
| Random Forest (RF) | mtry =4, min.node.size=1, splitrule="gini" | mtry: [1, 5], min.node.size: [1,10], splitrule: ["gini","entropy"] |
| Support Vector Machine (SVM) | sigma = 0.008521, C = 0.1823 | sigma: [10⁻³,10³] (log), C: [10⁻³,10³] (log) |
| K-Nearest Neighbors (KNN) | k = 4 | k: [1,20] (integers) |
| Extreme Gradient Boosting (XGB) | eta = 0.2, max_depth = 9, gamma =0.1, colsample_bytree= 0.8, min_child_weight= 0.9, subsample=0.5, nrounds=100 | eta: [0.01,0.3], max_depth: [3,15], gamma: [0,5], colsample_bytree: [0.5,1], min_child_weight: [0.5,2], subsample: [0.5,1], nrounds: [50,200] (integers) |
| Gradient Boosting Machine (GBM) | n_estimators = 300, max_depth = 9, subsample =0.8, colsample_bytree = 0.7 | n_estimators: [100,800], max_depth: [3,9], subsample: [0.5,1.0], colsample_bytree: [0.5,1.0] |
